# Industrialized GMP Production of CD34^+^ Cells (ProtheraCytes®) at Clinical Scale for Treatment of Ischemic Cardiac Diseases Is Feasible and Safe

**DOI:** 10.1007/s12015-022-10373-5

**Published:** 2022-04-14

**Authors:** Philippe Hénon, Marc Kowalczyk, Anne Aries, Christine Vignon, Guillaume Trébuchet, Rachid Lahlil

**Affiliations:** 1 CellProthera SAS, 12 Rue du Parc, Mulhouse, France; 2 Institut de Recherche en Hématologie et Transplantation, Hôpital du Hasenrain, 87 Avenue d’Altkirch, Mulhouse, France

**Keywords:** Regenerative medicine, Heart diseases, CD34^+^ cells, VSELs, Cell expansion, Industrialization, ProtheraCytes^®^

## Abstract

Regenerative medicine now needs to pass a crucial turning point, from academic research to the market. Several sources/types of cells have been experimented with, more or less successfully. CD34^+^ cells have demonstrated multipotent or even pluripotent capacities, making them good candidates for regenerative medicine, particularly for treating heart diseases. Strongly encouraged by the results we achieved in a pilot study using CD34^+^ stem cells in patients with poor-prognosis acute myocardial infarcts (AMIs), we soon began the development of an industrialized platform making use of a closed automated device (StemXpand^®^) and a disposable kit (StemPack^®^) for the large-scale expansion of CD34^+^ cells with reproducible good manufacturing practice (GMP). This scalable platform can produce expanded CD34^+^ cells (ProtheraCytes^®^) of sufficient quality that, interestingly, express early markers of the cardiac and endothelial pathways and early cardiac-mesoderm markers. They also contain CD34^+^ pluripotent cells characterized as very small embryonic-like stem cells (VSELs), capable of differentiating under appropriate stimuli into different tissue lineages, including endothelial and cardiomyocytic ones.

## Introduction

The ambitious goal of cell therapy/regenerative medicine is to structurally and functionally regenerate organs damaged by non-curable diseases, age or trauma, overcoming the problem of the critical lack of transplantable organs, which additionally require the long-life administration of immunosuppressive drugs, with their share of unwanted effects.

Theoretically, pluripotent embryonic stem cells (ESCs) are the best candidates for application in regenerative medicine. However, besides huge ethical considerations, their clinical use faces unresolved technical problems and the risk of teratomas and other tumors being formed. The development by Yamanaka in 2006 of induced pluripotent stem cell (iPSC) technology has been considered as an exciting alternative source of pluripotent stem cells, avoiding ethical issues. However, their unexpected genomic instability and variability, resulting in a risk of tumorigenesis and of the development of immunogenicity, hampers their clinical use [1]. At the same time, tissue resident pluripotent stem cells called very small embryonic-like stem cells (VSELs) were reported as responsible of tissues homeostasis and regeneration [2, 3]. VSELs are cells of small size (5 to 6 μm in diameter), deposited during ontogenesis and residing for life in the BM [4]. They represent a rare population (0.01% of BM-MNCs), phenotypically lineage-negative (Lin^−^), CD45^−^ CD34^+^ and/or CD133^+^ [5, 6]. They are able, in in vivo experimental models, of contributing to angiogenesis and myocardial repair representing a real alternative to ESCs and iPSC for regenerative medicine [7, 8].

However, to date, so-called “adult stem cells” have mainly been used for regenerative medicine. Most attempts have essentially concerned heart diseases. But most trials have been performed in academic centers in an experimental manner that cannot be replicated on a large scale. CD34^+^ stem cells (SCs) appear to emerge as the most convincing cell type among those that have been experimentally and clinically evaluated [9]. They are identified via the CD34 antigen, discovered by Civin et al. [10] and have long been considered as solely hematopoitic stem cells (HSCs). However, in 1997, Asahara et al., demonstrating that endothelial progenitor cells also bore the membrane CD34 antigen, opened the door to new investigations into the existence of potential non-hematopoietic CD34^+^ SC subpopulations [11]. Data from experimental AMI studies performed in SCID-nude mice showed that human CD34^+^ SC engrafted in the ischemic area and that their progeny differentiated into cardiac and endothelial lineages, which correlated with cardiac-function improvement [12, 13].

On the end of 2002, we launched a pilot study using autologous peripheral blood (PB)-CD34^+^ SCs, collected by leukapheresis (LKP) after granulocyte-colony-stimulating-factor (G-CSF) mobilization and then immuno-selected, in patients with poor-prognosis AMI scheduled for compassionate coronary artery bypass graft (CABG) operations not reperfusing the ischemic area [14]. The short- and long-term outcomes were very consistent, with an average progressive increase in left ventricle ejection fraction (LVEF) of 21 points from the baseline values 48 months after the procedure, and sustained structural and functional scar repair demonstrated by PET-scan imaging and 3D echography.

However, the procedure we used was difficult and demanding for the patient and could only have been performed in a select few specialized centers, which would have limited access to the therapy to a small number of AMI patients. Furthermore, regulatory authorities now consider CD34^+^ cells used for cardiac repair, or other non-hematopoietic indications, an advanced therapy medicinal products (ATMP).

A robust process that could allow the reproducible (GMP)-compliant production of CD34^+^ cell grafts was thus necessary to further improve patient access. We decided to simplify and standardize the CD34^+^ SC production process by developing an automated device that would allow GMP stem-cell expansion in vitro from whole blood (WB) samples withdrawn after G-CSF mobilization, to yield CD34^+^ SC numbers at least equivalent to those collected during one LKP procedure, thus avoiding the need for the latter.

The concept of regenerative medicine now lies at a crossroads, as it has to pass a crucial turning point to reach the market, i.e., the development of industrial tools allowing the good manufacturing practice (GMP)-observant, reliable and stable production of cell grafts, now considered as biotherapies.

## Summary of GMP Manufacturing Process of CD34^+^ SC (ProtheraCytes^®^)

The full project of developing a platform for the automated production of cells at a clinical scale was defined in order to fulfil two crucial factors, i.e., obtaining purified CD34^+^ SCs in a large number while starting from a single patient’s WB sample. The manufacturing process has been previously described [15]. Briefly, it includes: 1) the isolation of total nuclear cells from a WB sample after CD34^+^ cell mobilization by a daily sub-cutaneous (sc) injection of 10 μg/kg of G-CSF for five days; 2) CD34^+^ immunoselection; 3) a nine-day CD34^+^ cell expansion process; 4) cell-culture recovery in an aseptic closed system kit within the device, followed by 5) expanded-CD34^+^ SC immunoselection and conditioning. The final ATMP product is called ProtheraCytes^®^, the characteristics of which have been defined from whole blood samples harvested in healthy donors, as follows: expanded CD34^+^ SC count ≥10 × 10^6^; cell viability ≥95%; cell purity ≥80%; monocytes ≤15%; granulocytes ≤5%; lymphocytes B + T + NK ≤ 3% [15]. The ProtheraCytes^®^ demonstrated positive outcomes: no chromosomal alterations, no toxicity, no tumorigenicity, low biodistribution and similarity in genetic properties between ProtheraCytes^®^ and native CD34^+^ SCs. Similar numbers of CD34^+^ epitopes on the cell surface and similar telomere lengths in native and expanded CD34^+^ cells demonstrated that the cell stemness was not impaired by the expansion process, which multiplied the number of CD34^+^ SCs by 19.1- fold ±7.5-fold.

## Materials and Methods

### AMI Patients’ Blood Harvest Samples

A batch of 220 ± 10 ml WB was harvested by simple venous puncture on the sixth day morning following a five-day CD34^+^ SC mobilization by G-CSF (10 μg/kg/day S.C.) in 14 AMI patients enrolled in an on-going phase I-IIb clinical trial approved by EMA, ANSM, and MHRA (EUDRACT 2014–001476-63), scheduled to evaluate, primarily, the safety and, secondarily, the efficacy of the intracardiac administration of ProtheraCytes^®^, versus the standard of care (SOC) in patients that have undergone severe left-ventricular AMI (LVEF<50% at baseline) and are at high risk of developing secondary chronic heart failure (CHF). The batches were immediately shipped at 4 °C–10 °C from the participating clinical sites to one of our three production centers (two in France, one in UK) for CD34^+^ cell manufacturing along the process developed and validated during the healthy donors program. All patients signed informed consent.

### Healthy Donors and Controls

Frozen samples of Purified CD34^+^ SC (native CD34^+^) and expanded cells (ProtheraCytes^®^) were obtained from 7 healthy donors having been enrolled in the study published by Saucourt et al. in [15]. As this study is ended and the volunteers blood access is very limited, frozen CD34^+^ cells from 4 healthy donors commercialized by Lonza (NC, USA), isolated from bone marrow mononuclear cells by positive immunomagnetic separation were also additionally proceeded under automated GMP cell expansion and used as controls for all flow cytometry studies (FCM).

### ProtheraCytes^®^Analyses

#### Total CD34^+^ Cell Enumeration

CD34^+^ SC counts were performed at each stage of the manufacturing process, using the Stem Cell Enumeration kit and the Stem Cell Control kit (both from BD Biosciences, San Jose, CA) and analyzed with a Fluorescence Activated Cell Sorting (FACS) Canto II analyzer (BD Biosciences) and FACS DIVA software [16].

#### Graft Acceptance Criteria

CD34^+^ cell counts, cell viability, graft purity, sterility tests, Gram staining, and mycoplasma and endotoxin statuses were systematically determined - according to the specifications previously determined in [15] on each ProtheraCytes^®^ batch to assess the graft acceptance criteria through final quality controls.

#### Markers of ProtheraCytes^®^

##### Endothelial and Cardiac Differentiation

The expression of antigens associated with cardiomyocyte and endothelial lineages was evaluated in the AMI and controls with the following mouse anti-human monoclonal antibodies surface markers: APCVio770 anti-CD45 (Miltenyi Biotec, Clone5B1), FITC anti-CD34 (Miltenyi, clone AC136), APC anti-CD31 (Miltenyi Biotec, cloneAC128), PE-Vio770 anti-CD133 (Miltenyi Biotec, clone AC133), VB anti-CD105 (Miltenyi Biotech, clone A3A4E), PE anti-CD73 (cloneAD2, Miltenyi Biotec), PE-Vio770 anti-CD117 (Miltenyi Biotec, clone A3C6E2). Cell viability was monitored by the absence of dye 7-AAD uptake (BD Biociences). The cells were then analyzed using a FacsCanto II (Becton Dickinson Biosciences, Grenoble, France).

##### ProtheraCytes^®^ Transcriptome Analysis

Total RNA was purified from CD34^+^ SC (native CD34^+^) and ProtheraCytes^®^ collected in 8 AMI patients and 7 healthy donors using RNAeasy plus minikit (QIAGEN, Courtabeuf, France). Briefly, sample quality was assessed using a DNA High sensitivity chip (Agilent Technologies). Single-end 50 reads barcoded RNA-Seq sequencing library was performed on an Illumina HiSeq4000 with the TruSeq SBS v3 chemistry at iGE3 Genomics Platform (University of Geneva, Switzerland). The normalization and differential expression analysis was performed with the R/Bioconductor package edgeR v.3.28.1, for the genes annotated in the reference genome. Lowly expressed genes were filtered, keeping genes that are expressed at a reasonable level (10 counts in at least 7 samples). The filtered data were normalized by the library size. The differentially expressed genes were estimated with the GLM approach (Generalized Linear Model) using a negative binomial distribution. The genes were considered as differentially expressed when the fold change (FC) was at least 2-fold with a 5% false discovery rate (FDR) for multiple testing corrections according to Benjamini-Hochberg.

##### VSELs Quantification

To assess the presence of VSELs, ProtheraCytes^®^ and their native cells taken from Controls or AMI patients were stained with a mixture of lineages (Lin) associating monoclonal antibodies (MoAbs) conjugated with fluorescein isothiocyanate (FITC). At the same time, V500 conjugated-CD45 (Beckman Coulter), CD34 PE clone 8G12 and a combination of allophycocyanin (APC) conjugated MoAbs, CD133 clone AC133 (Miltenyi Biotec, Paris, France), were added for 30 min on ice. The labelled cells were washed and discriminated by FCM on the basis of cell size, granularity, absence of Lin and CD45 markers and presence of CD34 and CD133. The FCM analysis was performed using a BD ARIA III instrument (BD Biosciences). Data acquisition and analysis were conducted using BD-FACSDiva software (BD Biosciences).

### Statistical Analyses

Statistical analyses were performed using GraphPad Prism version7.03 for Windows (GraphPad Software, La Jolla, CA) and R (version 3.0.2, http://cran.r-project.org/) software. All values are expressed as the mean _ SD. After checking whether the data had a normal distribution (Shapiro–Wilk test), Student’s t test or non-parametric tests (Kruskal–Wallis or Mann–Whitney) were used to compare the native CD34^+^ and eCD34^+^ SC groups. *p* < .05 was considered to be significant.

## Results

### CD34^+^ SC GMP Production Platform

**Fig. 1:**
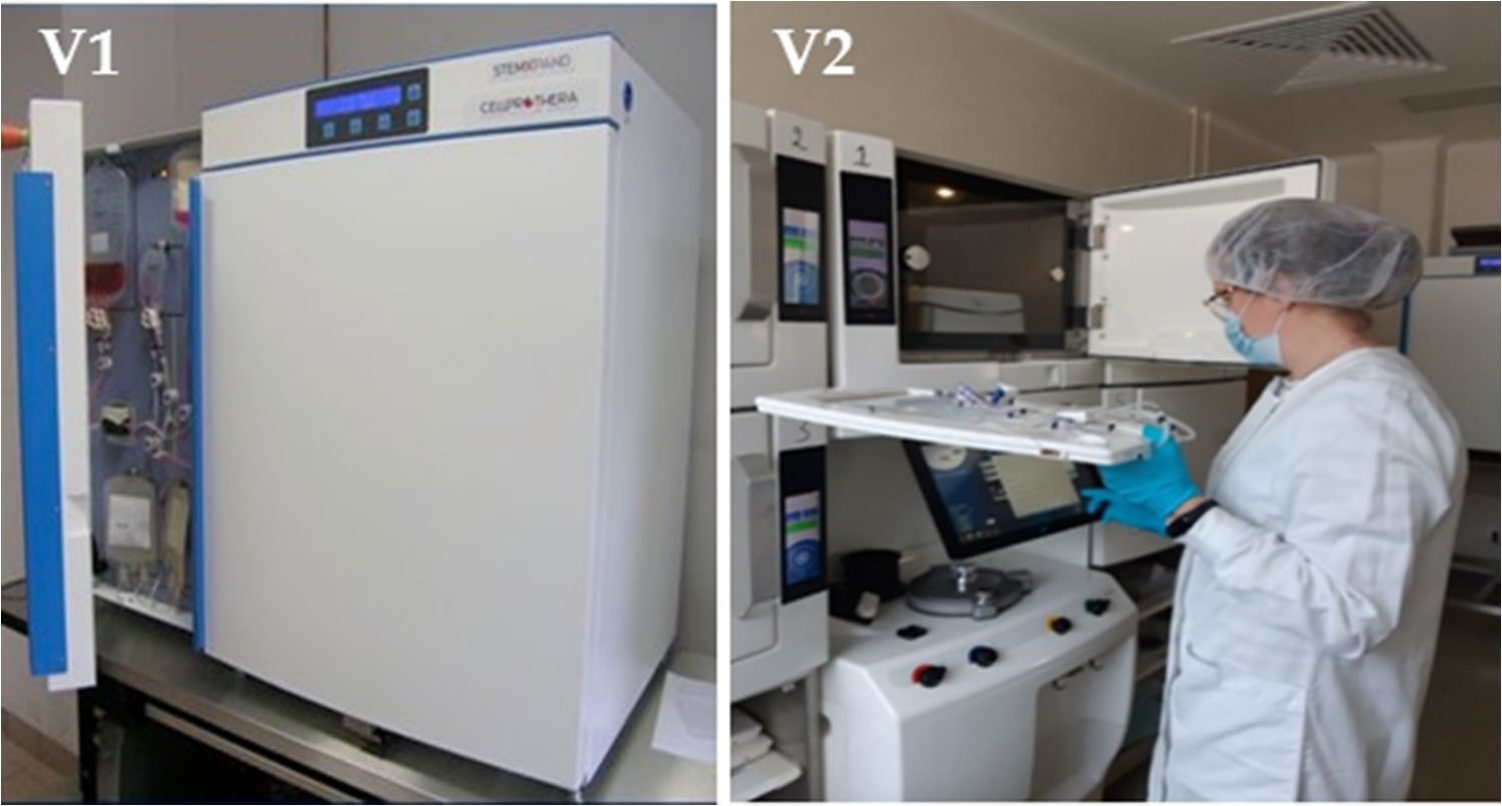
The StemXpand^®^ cell expansion platform: V1 clinical version with one incubator and production kit (left); and the V2 clinical version (on process) comprising 5 incubators and an aseptic cassette replacing the previous kit of production (right)

The platform we have developed allows for the reproducible, automated and standardized production of the ProtheraCytes^®^, which have been authorized as an investigational medicinal product (IMP) by the EMA and the regulatory agencies in France and the UK (ANSM and MHRA). The proprietary cell-expansion platform (V1) comprises an automated device (StemXpand^®^) and a disposable production kit StemPack® (Fig. 1). The automated production system is patent-protected globally [16] and is ISO 13485 certified.

### AMI Patients’ ProtheraCytes^®^ Characterization

#### ATMP Specifications

The ProtheraCytes^®^ obtained from 14 AMI patients out of the 33 scheduled who have been presently enrolled in the ProtheraCytes^®^ group of the EXCELLENT study have been cautiously analyzed. The average number of CD34^+^ SCs obtained after a 9-day expansion process is 142.9 × 10^6^ (range: 35.4–277.8 × 10^6^), corresponding to a 16.4-fold expansion rate (range: 4.2–29.4). Beside CD34^+^ SCs, ProtheraCytes^®^ also contained, on average, 3.5% monocytes, 0.8% granulocytes and 0% lymphocytes. Other data obtained from the final ProtheraCytes^®^ product, i.e. viability, purity, and the sterility tests, were in accordance with the previously established specifications [15].

#### Endothelial, Mesoderm and Cardiac Differentiation Genes Profiling

**Fig. 2:**
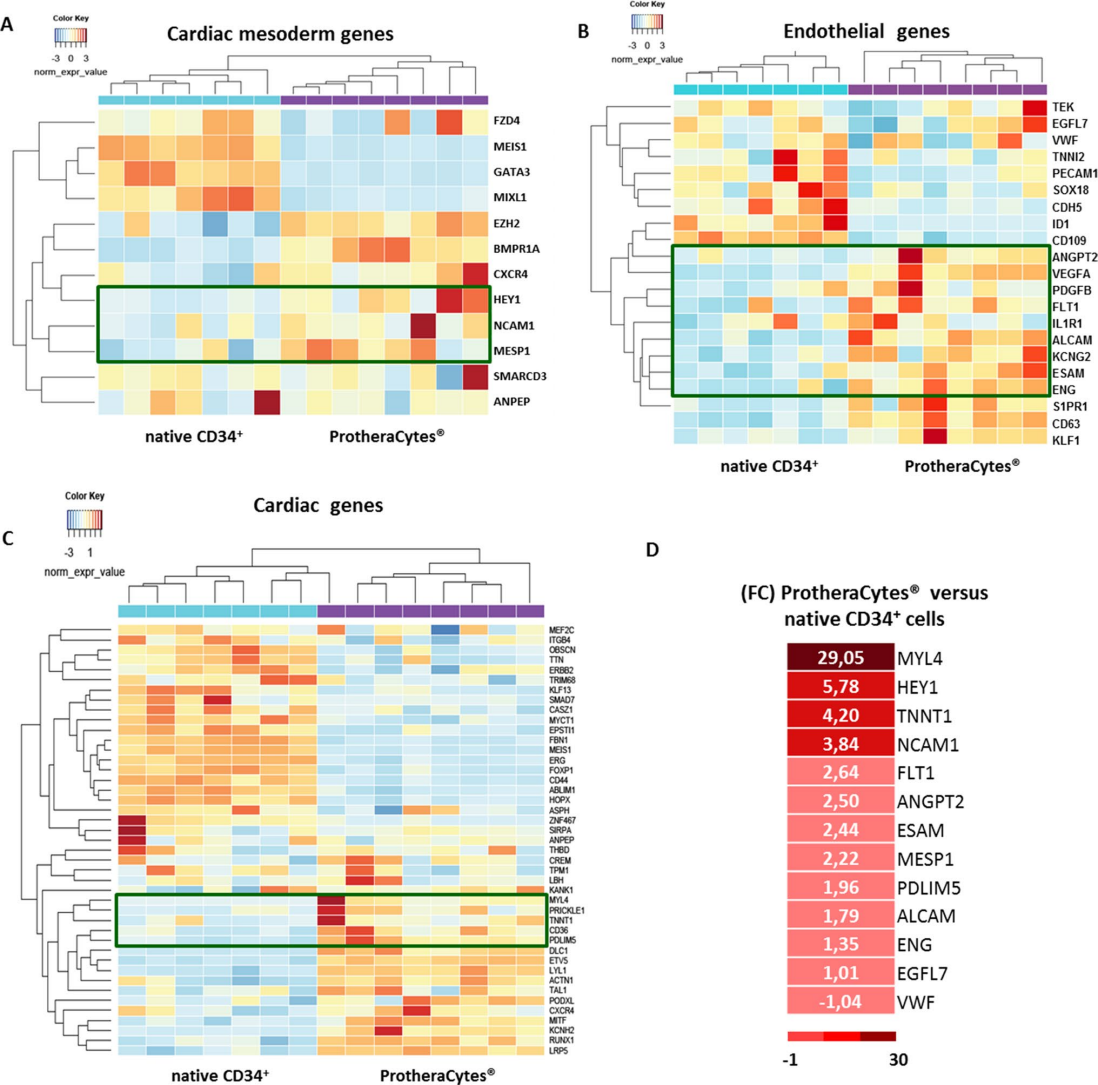
Heat map representing the differential expression of cardiac and endothelial very early markers detected in ProtheraCytes^®^ versus native CD34^+^ cells at day 0 from AMI patients at day 0. Genes are grouped into three categories, including mesoderm, cardiac mesoderm markers (A) and endothelial markers (B) and cardiac markers (C). Green boxes (A, B, C) highlight HEY1, NCAM1, MESP1, ANGPT2, FLT1, ESAM, ALCAM, MYL4, TNNT1 and PDLIM5. Native CD34^+^ SC are represented in blue (n = 7), the ProtheraCytes^®^ samples are in purple (n = 8). (D) Heatmap showing the main cardiac, mesoderm and endothelial genes induced in ProtheraCytes^®^ by comparison to CD34^+^ cells expressed as fold change (FC); the color scale is represented at the bottom

Interestingly, by using RNA sequencing study, the differential expression of cardiac and endothelial very early markers detected in ProtheraCytes^®^ versus native CD34^+^ cells from AMI patients at day 0 showed that these very early markers implicated in the cardiovascular pathways are expressed at the molecular level, and some of them highlighted in the green boxes were significantly induced (Fig. 2A). They enclose the markers of mesoderm and cardiac mesoderm progenitors such as NCAM1 (neural cell-adhesion molecule) with 3.84 fold change (FC) in ProtheraCytes^®^ versus native CD34^+^ [17], MESP1 (mesoderm posterior bHLH transcription factor 1) (2.22 FC) [18], HEY1 (hes-related family bHLH transcription factor with YRPW motif 1) (5.78 FC) (Fig. 2A, D). Similarly, endothelial markers (Fig. 2B) such as, FLT1 (fms related tyrosine kinase 1) known as VEGFR1 (vascular endothelial growth factor receptor-1) - previously reported to play a vital role in regulating vascular biological function - [19], as well as ANGPT2 (angiopoietin 2) and ESAM (endothelial cell adhesion molecule) were enriched significantly by a FC of at least >2 (Fig. 2D).

Along with genes improving angiogenesis, ALCAM (activated leukocyte cell adhesion molecule) [20] was also slightly upregulated in ProtheraCytes^®^ (1.79 fold). In addition, the expression of cardiac genes including, TNNT1 (troponin T1, slow skeletal type), PDLIM5 (PDZ and LIM domain 5) [21] and MYL4 (myosin light chain 4) was enhanced (Fig. 2C). In contrast, other endothelial markers such as VWF and EGFL7 are expressed but not induced in AMI ProtheraCytes^®^. The FC expression of these genes in ProtheraCytes^®^ when compared to native CD34^+^ stem cells seeded at day 0 are summarized in fig. 2D.

**Fig. 3:**
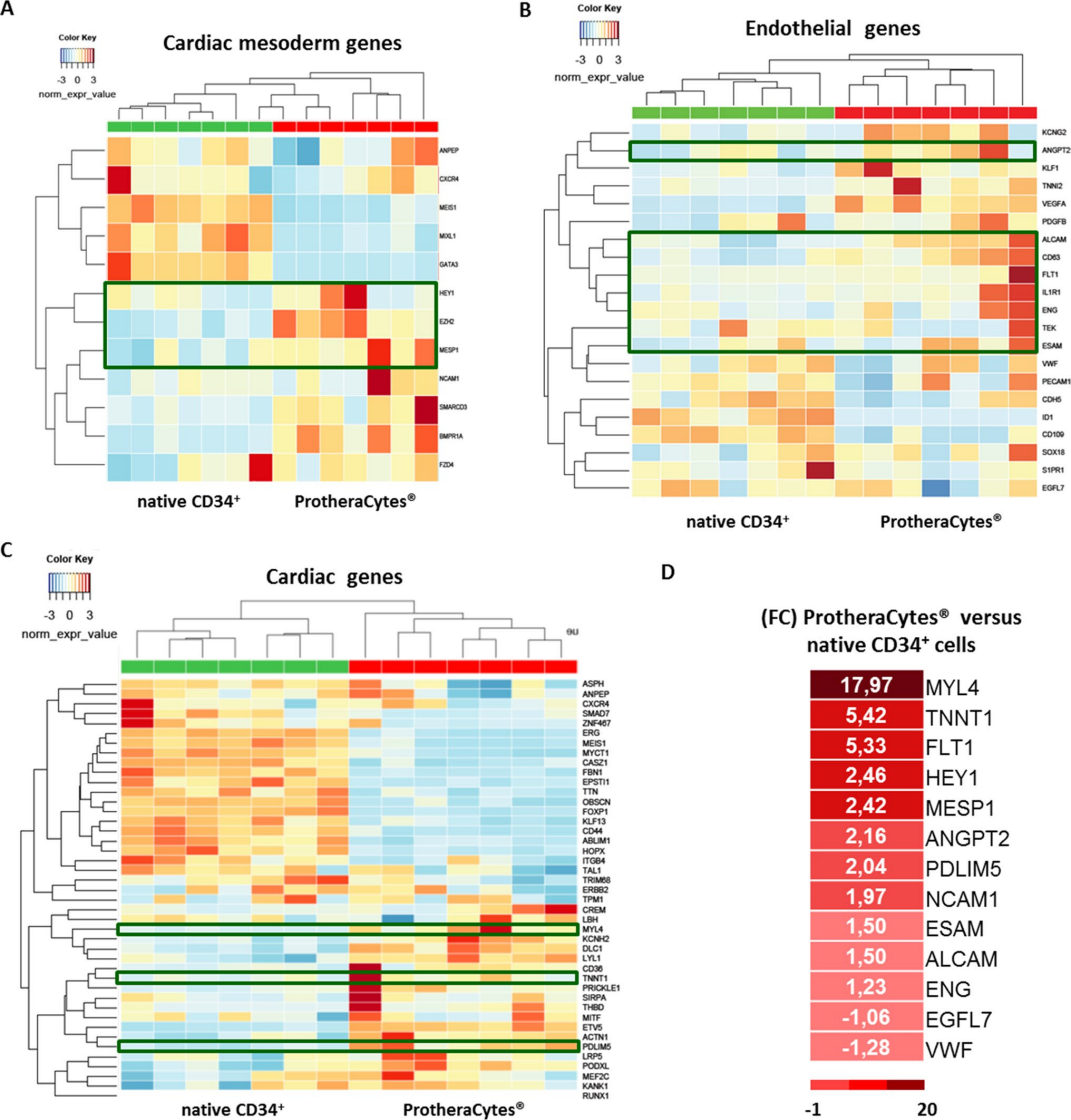
Heat map representing the differential expression of cardiac and endothelial very early markers detected in ProtheraCytes^®^ versus native CD34^+^ cells at day 0 from Healthy donors at day 0. Genes are grouped into three categories, including mesoderm, cardiac mesoderm markers (A) and endothelial markers (B) and cardiac markers (C). In (D) Heatmap showing the main cardiac, mesoderm and endothelial genes induced in ProtheraCytes^®^ by comparison to native CD34^+^ cells expressed as fold change (FC); the color scale is represented at the bottom

Finally, as shown in fig. 3, the differential expression of cardiac, very early endothelial markers and cardiac genes in ProtheraCytes^®^ versus native CD34^+^ cells at day 0 from healthy donors indicates also that these genes groups are expressed and that some of them were upregulated in ProtheraCytes^®^ even to lesser extent than seen in AMI patients (Fig. 3).

**Fig. 4:**
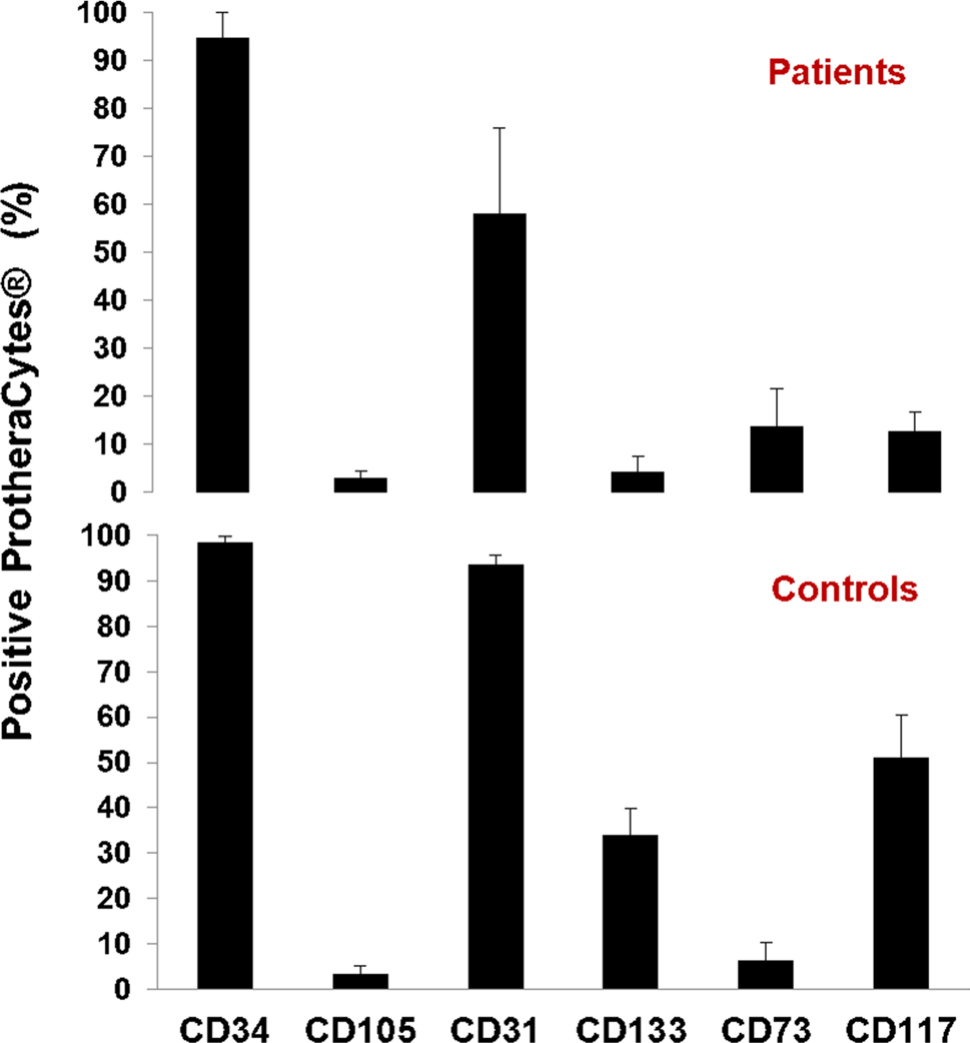
The stem cells and endothelial progenitor cell markers detected in ProtheraCytes^®^ by flow cytometry from AMI patients (n = 12) and controls (n = 4)

On the other hand, according to FCM analyses, these cells expressed CD31 (58% vs 93.4%), CD73 (13.6% vs 6.4%), CD117 (12.6% vs 51%), CD105 (2.9% vs 3.3%), and CD133 (4.1% vs 33.9%), in AMI patients as well as healthy donors ProtheraCytes^®^ respectively suggesting that they were still able to express surface markers of both stem cells and endothelial progenitor cells (Fig. 4). PCR and FCM studies are still in progress to confirm these data at a larger scale.

#### VSELs Quantification

**Fig. 5:**
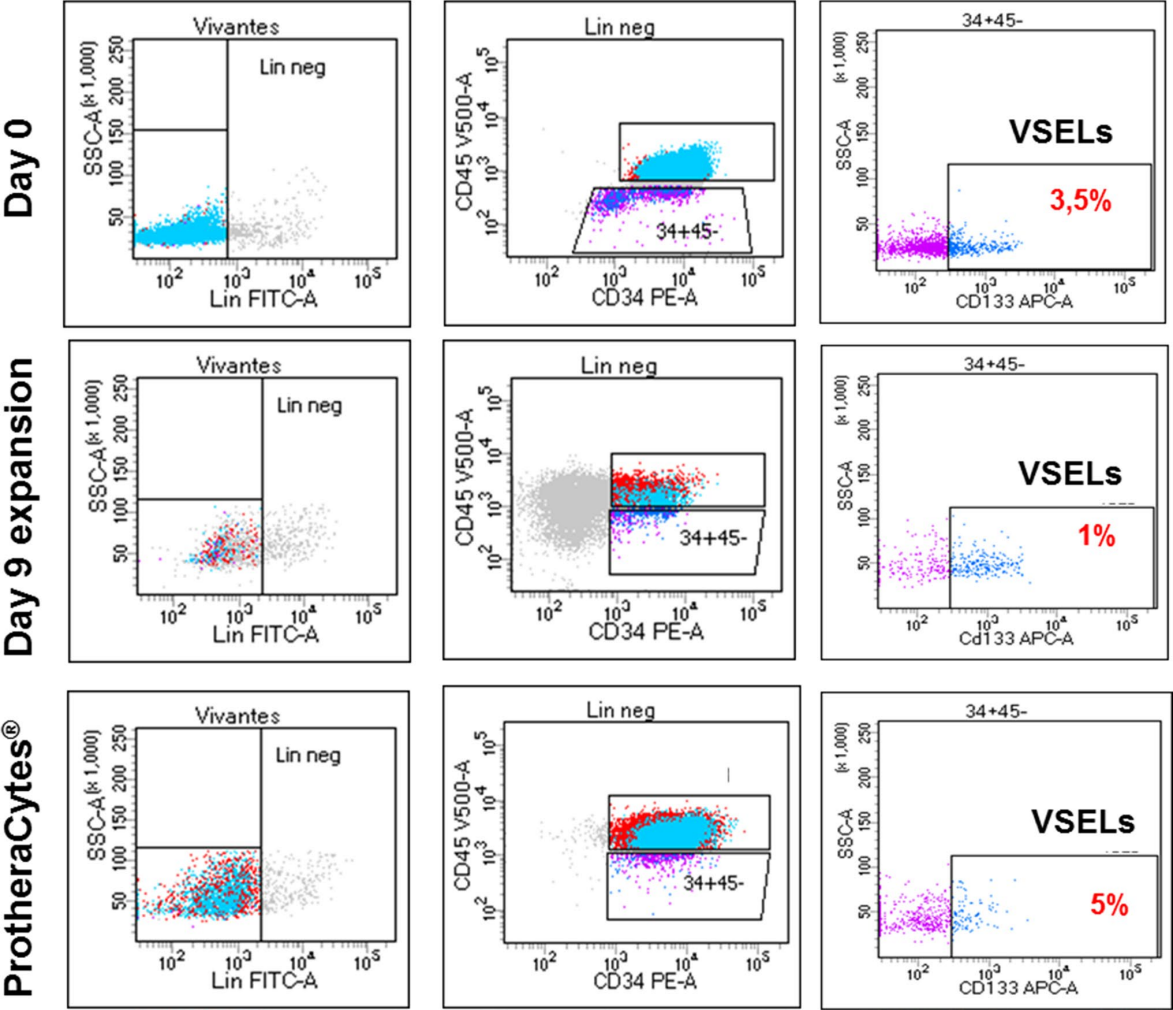
Representative experiment showing, the Lin^−^CD34^+^CD45^−^CD133^+^ VSELs presence in control on day 0, in day 9 expanded CD34^+^ SC before immunoselection and in purified ProtheraCytes^®^ as detected by flow cytometry

**Fig. 6:**
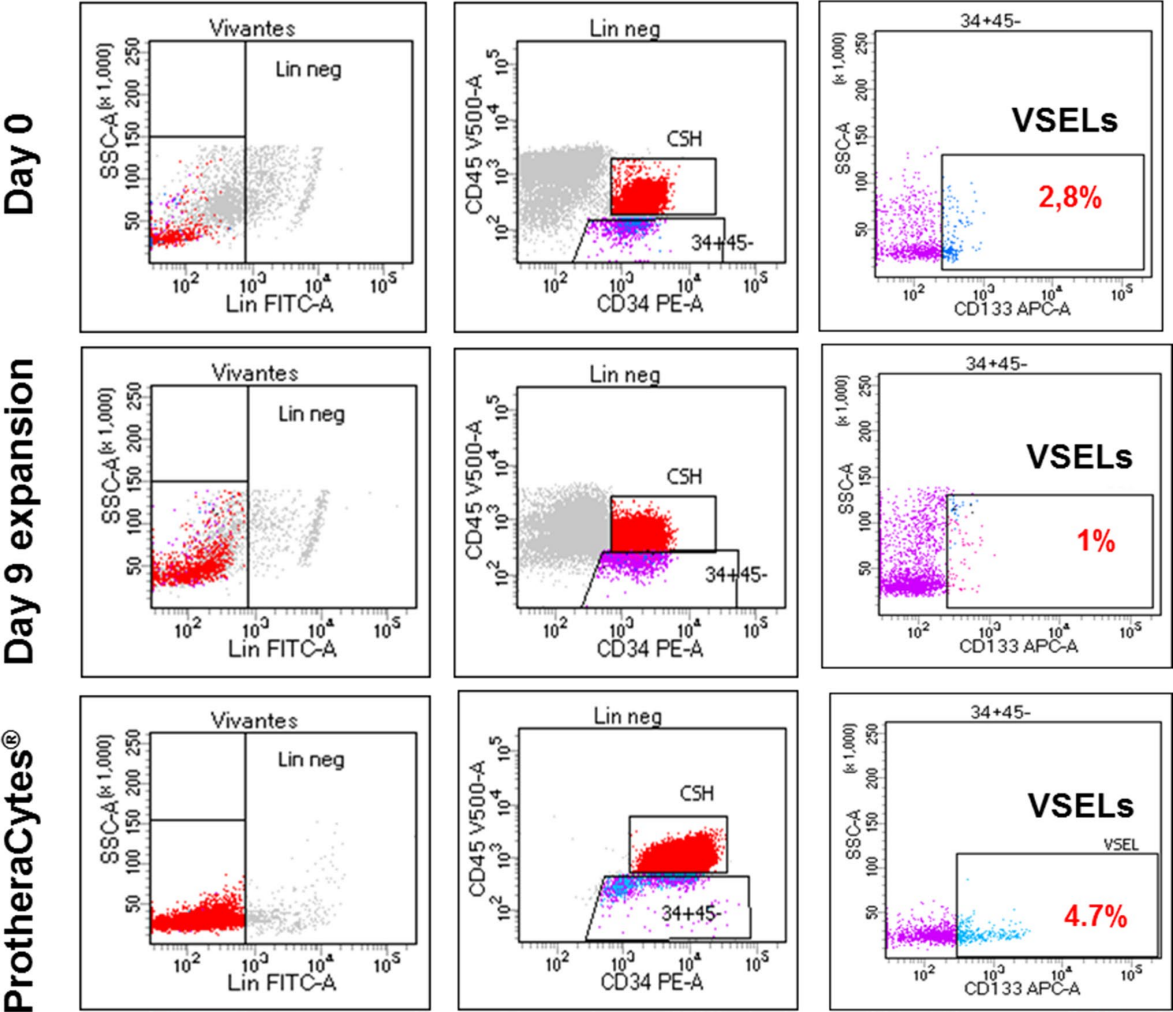
Representative experiment showing, the Lin^−^CD34^+^CD45^−^CD133^+^ VSELs presence in AMI patients on day 0, in day 9 expanded CD34^+^ SC before immunoselection and in purified ProtheraCytes^®^ as detected by flow cytometry

On day 0 we detected by FCM around 3.5% of VSELs relative to lineage negative cells among the controls immuno-selected CD34^+^ SC (Fig. 5, upper panel). Once expanded for 9 days, VSELs represented 1% and finally after immuno-selection and purification of the ProtheraCytes^®^ VSELs represented around 5% (Fig. 5 middle panel and lower panel respectively). Taken into account that CD34^+^ SC were expanded for 9 days to obtain ProtheraCytes^®^, an average of 4 to 5 × 10^6^ CD34^+^ cells seeded on day 0 containing 5% of VSELs of the total were obtained from each 4 to 5 × 10^5^ ones comprising around 3.5% VSELs of the total, which represent at least a 10 fold expansion. The data in fig. 5 represent results from controls (a representative experiment from n = 4), nevertheless, we didn’t observed significant differences in the VSELs expansion rate when CD34^+^ SC or ProtheraCytes^®^ were obtained from controls (Fig. 5) or from patients (Fig. 6), suggesting that the VSELs pool is not significantly affected by the occurrence of AMI.

## Discussion

The future of any biotherapy now requires to develop a GMP-compliant and industrialized bioproduction process that includes several challenges that had not been previously identified within the known scopes of the centralized manufacturing of pharmaceutical molecules. For autologous cell therapies, they are various, as a cell is a more complex working material than molecules and differs from patient to patient. These challenges include manufacturing close to the patient’s “bedside” in decentralized, scalable and validated manufacturing centers with qualified and regularly trained staff that permit easy access to the treatment, since a rapid transport of fresh raw material and the final graft between the manufacturing center and the hospital are involved. The current regulatory status requires the manufacturing center to be compliant with the GMP standards and include cleanroom environments to guarantee aseptic processes and perfect traceability. Each step must be characterized and repeatable since each production center must deliver the same product characteristics and not only comply with the final product specification, but also be validated within comparability studies. Such a manufacturing process shall then allow the degree of automation to be maximized, a modularity that allows scalability, and compatibility with decentralized manufacturing.

In response to those challenges and the constantly evolving regulatory framework, we developed, between 2011 and 2020, a complete industrial solution for CD34^+^-graft production on a large clinical scale (V1; Fig.1, left).

The CD34^+^ SCs expansion-fold achieved after expansion in the EXCELLENT trial’s patients via this platform is a little lower than those observed in healthy volunteers [15], but not significantly so (16.4 vs 19.1 *p* > *0.5*). This is related to larger individual variations in cell mobilization/expansion capacity, due to the frequent co-morbidities presented by many of these patients (diabetes mellitus, smoking, elderly patients, etc.), which are known to have negative impacts on CD34^+^ mobilization by G-CSF [22, 23].

Thus, the V1 original automated process has been proven to be robust and reliable through mechanical and biological validations. However, it did not yet include a modular approach, allowing only 2–3 cell expansions per month. This limitation was addressed within a new version of the StemXpand^®^ platform called V2 (Fig. 1 right). The improvements focused on the scale-up of the manufacturing capacity—which, with five independent modules, was increased to 15 cell expansions per month—and on its modularity, as the manufacturing center can host one, three or five incubator modules. The aseptic kit is much simpler to handle and includes RFID tags for traceability, security and the proprietary use of the platform–kit duo, and the incubator can also uniformly thaw the frozen culture medium if necessary. A second PCT patent family was filed for the unique electromechanical inventive solutions found and applied [22].

The promise of CD34^+^SC therapy for various therapeutical indications, including acquired cardiac diseases, raises the question of the specific identity of the progenitor cells that would affect organs repair. The “true” HSC population only represents a small proportion (~ 1%) of the total CD34^+^ cells [24]. It has been progressively established that the CD34 antigen is also a marker of endothelial [11], cardiac [25, 26], osteoblastic and cartilage [27], liver [28], and likely other progenitor cells, each of them not representing more than 1% of the total CD34^+^ population.

In fact, the intensity of CD34 expression on the cell’s membrane is heterogeneous and correlates with the stage of immaturity/maturity, subdividing cells into CD34^bright^ and CD34^dim^ [29]. The earliest tissues’ progenitor cells are members of the CD34^bright^ group. We had previously demonstrated that G-CSF-mobilized CD34^+^ cells included cells featuring immunophenotypic and gene characteristics of both endothelial and cardiac-muscle progenitor cells. Additionally, in vitro culture of still-undifferentiated CD34^bright^ cells in a specific and proprietary MV06™ medium induced the further development of adherent cells co-expressing characteristics of endothelial (VEGFR-2/CD133) or cardiac-muscle (c-Troponin-T and sarcomeric-α-actin) lineages, suggesting the initiation of endothelial- and cardiac-muscle-cell differentiation pathways [14]. By contrast, CD34^dim^ mainly co-express CD133/CD45, characterizing them as granulocytic progenitor cells [29].

In the present study, we reported the identification of markers that were described in the literature as being proven that their expression allows the isolation of cells capable of simultaneously generating endothelial cells and cardiomyocytes and therefore enhancing cardiac function potential. Indeed, ALCAM^+^ cells are known to restore cardiac function after their transplantation in an in vivo rat model of myocardial infarction [20], these cells are able to generate multiple cell types including cardiomyocytes and endothelial cells. Indeed, transplantation of mesodermal cardiovascular progenitors, generating both vascular cells and cardiomyocytes has been shown in a nude rat model to improve cardiac function [30]. Endothelial progenitor cells where angpt2 is upregulated appear to have enhanced angiogenesis when administered to a syngeneic rat model of AMI. MYL4 expression is robust and homogeneous in the atria and is commonly present in fetal and neonatal ventricular tissues [31] and its expression like that of Mesp1 are required for heart morphogenesis and vasculature development [32]. The expression of such genes on ProtheraCytes^®^ comforts their potency for cardiac repair.

The identification of VSELs presence among ProtheraCytes^®^ could enhance their cardiac repair as studies have demonstrated through animal models that VSELs are capable of regenerating cardiomyocytes, vascular and endothelial cells [33–35]. In addition, VSELs are actively mobilized from the bone marrow into the peripheral blood following MI, contributing to the repair of the infarcted myocardium [36]. VSEL-derived cells show vasculogenic potential, as they trigger post-ischemic revascularization in immunodeficient mice and acquire an endothelial phenotype either in vitro or in vivo [37].

Furthermore, the number of VSELs present in ProtheraCytes^®^ purified either from healthy donors or patients does not appear to be significantly different, which is consistent with the fact that VSELs number remains stable in blood and heart throughout life despite the advanced age and associated comorbidities [38–40]. However, they can be mobilized in larger numbers, by G-CSF, from the BM into the PB [41] and expanded ex vivo [8, 42]. Our present data showed that GMP automated manufacturing process we have developed seem to also expand accurately the CD34^+^ VSELs among ProtheraCytes^®^ as we described previously for CD34^+^ cord blood SCs [42]. It has also recently been shown that expanded VSELs can efficiently give rise to endothelial colony-forming cells when stimulated by nicotinamide acid [43] allowing to believe in the ProtheraCytes^®^ capacities in cardiac repair. Then, a comparable effect was observed in a congestive heart failure model [44]. Thus, CD34^+^ VSELs isolated from adult tissues appear to be “true” pluripotent stem cells, which could be used, through their progeny, to regenerate a damaged heart. They could be at least partly responsible for the repair and improvement of cardiac function observed when using CD34^+^ cells in regenerative medicine after AMI. Here, interestingly, healthy donors and AMI patient’s ProtheraCytes^®^ contain both substantial numbers of multipotent stem cells of the endothelial and cardiac pathways, characterized at the molecular level and, to a lower degree, by FCM, and pluripotent VSELs. Combined, these cells at different stages of non-commitment/commitment are probably able to induce and sustain cardiac repair.

**Fig. 7:**
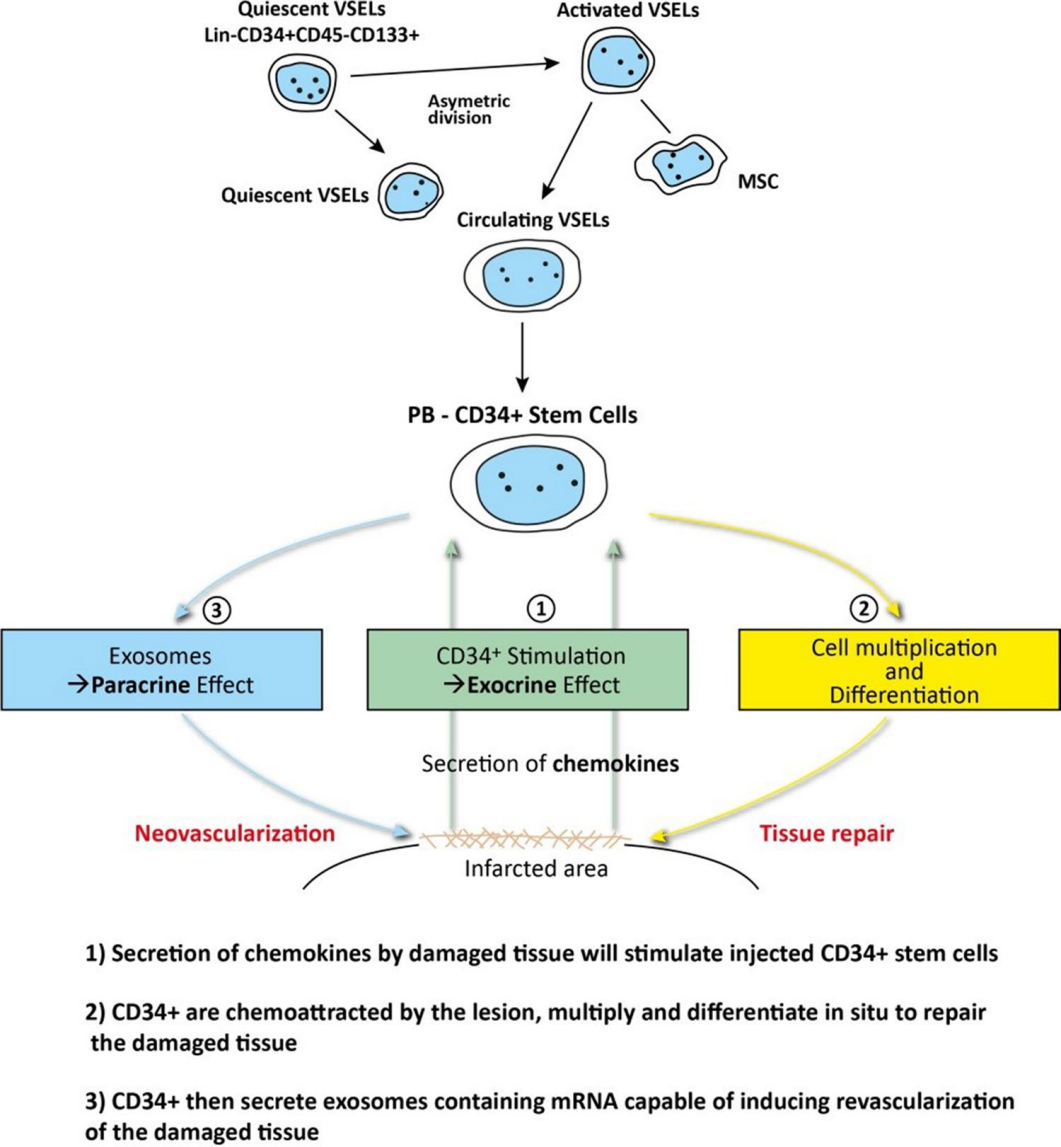
Proposal for the different biological mechanisms involved in CD34^+^ stem cell cardiac regenerative medicine

The underlying cardiac-repair mechanisms of VSEL-derived CD34^+^ subpopulations are probably multi-faceted (Fig. 7). First, a complex blend of cardioactive chemokines secreted by the inflammatory scar [45, 46] chemoattracts the injected CD34^+^ SCs to home in on the ischemic zone and induces their commitment along the endothelial and cardiac pathways [47]. Then, once activated by the scar chemokines, CD34^+^ SCs may release soluble paracrine factors and exosomes that can enhance the proliferation of resident cardiomyocytes [7, 8] or support neoangiogenesis [48], respectively, thus reducing fibrosis and attenuating remodeling effects. These cellular and molecular events are strongly dependent on changes in myocardial stiffness that occur after AMI [49]. Such commitment is crucial for the induction of cardiac tissue repair after ischemic heart disease. Furthermore, the hypoxic environment of the infarct zone increases vascular endothelial growth factor (VEGF) expression by transplanted cells, which may accelerate the proliferation of endothelial cells and α-SM actin-positive cells, as reviewed in [50].

## Conclusions

CD34^+^ cells might be the most convincing type of cells for regenerative medicine, particularly for cardiac repair after AMI, due to their specific biological properties. Their industrial GMP-compliant production in large numbers is feasible and safe and does not alter their biological/functional characteristics, thus fulfilling regulatory requirements and facilitating their extensive clinical use. More particularly, the ProtheraCytes^®^ contain both pluripotent VSELs and CD34^+^ cells expressing early markers of the cardiac and endothelial pathways, making them very good candidates for sustained heart repair after AMI.
